# What is schizophrenia – symptomatology

**DOI:** 10.1017/S1092852924000622

**Published:** 2024-12-02

**Authors:** Joan M. Striebel

**Affiliations:** Semel Institute for Neuroscience and Human Behavior, University of California, Los Angeles, CA 90650, USA

**Keywords:** schizophrenia, positive symptoms, negative symptoms, disorganization, cognitive impairment, social cognitive impairment, functional outcome

## Abstract

Schizophrenia is a highly heterogenous disorder with substantial interindividual variation in how the illness is experienced and how it presents clinically. The disorder is composed of primary symptom clusters—positive symptoms, negative symptoms, disorganization, neurocognitive deficits, and social cognitive impairments. These, along with duration, severity, and excluding other possible etiologies, comprise the diagnostic criteria for the disorder outlined in the two commonly used diagnostic classification systems—the Diagnostic Statistical Manual of Mental Disorders, Fifth Edition, Text Revision and the International Classification of Diseases, 11^th^ Revision. These primary symptoms as well as accessory symptoms (mood disturbances, anxiety, violence) and comorbidities (substance use, suicidality) bear upon each other to varying degrees and impact functional outcomes. The following review presents two patient cases illustrating the clinical heterogeneity of schizophrenia, the natural history of the illness and diagnosis, followed by the current understanding of the primary symptom clusters, accessory symptoms, and comorbidities. In addition to noting symptom prevalence, onset, and change over time, attention is paid to the impact of symptoms on functional outcome.

## Schizophrenia is clinically heterogeneous: patient-cases

Marta is a 65-year-old woman with schizophrenia. She suffered her first psychotic break at age 25 and has had multiple hospitalizations over the years. Her primary delusion is centered on the belief that she is a psychiatrist. She believes that medications she had taken in the past allowed the Central Intelligence Agency (CIA) to gain control of her mind and that the CIA sends people “mindreaders” to follow her. She refutes the diagnosis of schizophrenia and declines treatment. She’s been arrested multiple times for vandalism, terrorist threats, and assault. Although she’s been intermittently homeless, most recently, she was living at a motel, hit another guest whom she believed was sent by the CIA, and was arrested. In jail, correctional officers notice that she wears her clothes inside out, talks to herself, and when she talks to them, what she says doesn’t make sense.

DeShawn is a 23-year-old man with schizophrenia. He played sports in school, earned average grades, and had a large circle of friends. In high school, he began smoking cannabis and slowly withdrew from all activities. He suffered his first psychotic break at age 19 and was hospitalized for three months. While hospitalized, he felt so hopeless about the diagnosis of schizophrenia that he tried to hang himself in the bathroom. He attempted suicide again shortly after he was discharged. After spending several years recovering, he returned to community college. While his parents are thrilled with his progress, they worry that he spends so much time in his room, seldom talks with them, and has few friends. They miss the energetic, outgoing young man that he used to be and wonder if there’s a medication or something they can do to bring him back.

Marta and DeShawn illustrate the substantial variation in how individuals experience schizophrenia. The clinical complexity of the disorder manifests in different symptom domains, associated symptoms, comorbidities, disease trajectories, and in treatment response. The variation in the presentation and course of the illness was recognized when schizophrenia was first described over 150 years ago.

The two patient cases illustrate the core schizophrenia symptom clusters (Table 1). The diagnosis of schizophrenia is based on a combination of distinctive symptoms of sufficient duration and severity in the absence of other possible causes, e.g., substance use, medical or neurological illnesses, or other psychiatric illnesses. The diagnostic criteria for schizophrenia are outlined in the two diagnostic classification systems currently used in clinical practice—the Diagnostic Statistical Manual of Mental Disorders, Fifth Edition, Text Revision (DSM-5-TR) and the International Classification of Diseases, 11^th^ Revision (ICD-11) (Table 2). A key difference between the two systems is the DSM-5-TR’s inclusion of functional deficits as a criterion. This inclusion acknowledges the importance of neurocognitive and social cognitive impairments, symptoms that are largely responsible for the magnitude of disability in schizophrenia.^1^^-^^5^

**Table 1.** Schizophrenia Symptoms and Comorbidities

Primary Symptoms	Accessory Symptoms	Comorbidities
Positive symptoms	Mood disturbances	Substance use
Negative symptoms	Anxiety	Suicide
Disorganization	Violence	
Neurocognitive deficits		
Social cognition deficits

**Table 2.** Comparison of DSM-5-TR and ICD-11 Diagnostic Criteria for Schizophrenia

Subject of Comparison	DSM-5-TR Classification	ICD-11 Classification
Name of Chapter	Schizophrenia spectrum and other psychotic disorders	Schizophrenia and other primary psychiatric disorders
Nomenclature	Schizophrenia F20.9	Schizophrenia 6A20
Main Symptoms	Delusions Hallucinations Disorganized speech (e.g., frequent derailment or incoherence)	Persistent delusions Persistent hallucinations Disorganized thinking (formal thought disorder) Experiences of influence, passivity, or control
Diagnostic criteria	At least one main symptom is present for a significant portion of time during a 1-month period (or less if successfully treated).	At least one main symptom must be present (by the individual’s report or through observation by the clinician or other informants) most of the time for a period of 1 month or more.
Additional symptoms	Grossly disorganized or catatonic behavior Negative symptoms (i.e., diminished emotional expression or avolition)	Negative symptoms Grossly disorganized behaviour that impedes goal-directed activity Psychomotor disturbances such as catatonic restlessness or agitation, posturing, waxy flexibility, negativism, mutism, or stupor
Diagnostic criteria	If only one main symptom is present, at least one additional symptom is required.	If only one main symptom is present, at least one additional symptom is required.
Functionality criteria	For a significant portion of the time since the onset of the disturbance, level of functioning in one or more major areas, such as work, interpersonal relations, or self-care, is markedly below the level achieved prior to the onset (or when the onset is in childhood or adolescence, there is failure to achieve expected level of interpersonal, academic, or occupational functioning).	
Duration	Continuous signs of the disturbance persist for at least 6 months. This 6-month period must include at least 1 month of symptoms and may include periods of prodromal or residual symptoms. During these prodromal or residual periods, the signs of the disturbance may be manifested by only negative symptoms or by two or more symptoms present in an attenuated form (e.g., odd beliefs, unusual perceptual experiences).	Symptoms must be present most of the time for a period of 1 month or more.
Exclusions	Schizoaffective disorder, depressive disorder, or bipolar disorder with psychotic features The disturbance is not attributable to the physiological effects of a substance (e.g., a drug of abuse, a medication) or another medical condition. If there is a history of autism spectrum disorder or a communication disorder of childhood onset, the additional diagnosis of schizophrenia is made only if prominent delusions or hallucinations, in addition to the other required symptoms of schizophrenia, are also present for at least 1 month (or less if successfully treated).	Schizotypal disorder Schizophrenic reaction Acute and transient psychotic disorder The symptoms are not a manifestation of another health condition (e.g., a brain tumor) and are not due to the effect of a substance or medication on the central nervous system (e.g., corticosteroids), including withdrawal (e.g., alcohol withdrawal).

## Natural history of schizophrenia

**Figure 1.**
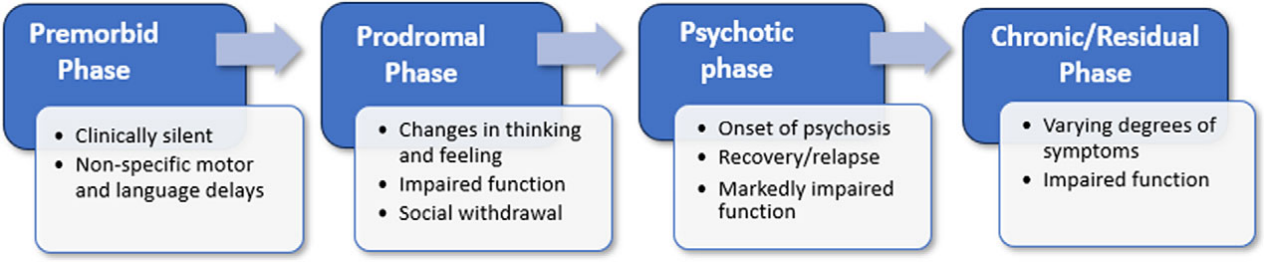
The natural history of schizophrenia.

For many individuals, schizophrenia follows a typical course that can be divided into four phases: premorbid, prodromal, psychotic, and chronic/residual (Figure 1).

## Premorbid stage

Children who go on to develop schizophrenia later in life are not prospectively distinguishable from their peers. If any abnormalities are present, they are subtle and nonspecific. Given schizophrenia’s neurodevelopmental origin, abnormalities in brain development manifest as early intellectual ^6^ ^-^^8^ and neuromotor abnormalities.^9^ Academic underachievement is often observed in elementary school and onwards in children who later develop schizophrenia compared with peers who do not go on to develop schizophrenia.^10^

## Prodromal stage

About 75-80% of people who develop schizophrenia experience a prodromal phase.^11^ For most patients, this stage lasts years and typically occurs in adolescence or early adulthood. Depressive symptoms are typically the first to emerge and appear on average 52 months prior to the first hospitalization.^11^ Other changes in thinking and feeling include anxiety, sleep disruption, and difficulties concentrating. Social withdrawal, role failure resulting in academic/occupational problems, and a decline in self-care are common. As the prodrome progresses, attenuated psychotic symptoms may develop. These include perceptual changes (colors may seem brighter or distorted, increased sensitivity to sounds), paranoia, uneasiness, preoccupation with certain ideas, and ideas of reference.

## Psychotic stage

This stage begins with the onset of frank psychosis, which may emerge abruptly over a period of days to weeks or insidiously over months or longer. The onset for men is in the early to mid-20s and for women in the late-20s or after age 40. Psychosis is characterized by the loss of touch with reality, altered perceptions, and can be profoundly distressing to the individual. Symptoms include hallucinations, delusions, and disorganized speech and behavior. The first psychotic episode is a crucial timepoint for intervention as patients tend to be more responsive to antipsychotics and psychosocial treatment. Delays in treatment result in a longer duration of psychosis which is associated with poorer outcomes in a variety of domains including increased severity of symptoms and decreased likelihood of remission.^12^ ^-^^14^

## Chronic/residual stage

There is considerable variation in the trajectory of the disease after the first psychotic episode, and many factors influence the course of the illness including genetics, gender, premorbid functioning, substance use, adherence to treatment, and physical health. After the first psychotic episode, about 20% of patients will recover as defined by clinical and social/functional recovery for at least one year.^15^ Others experience recurring psychotic relapses. Frequent and/or severe relapses can result in clinical deterioration or disease progression, which leads to decreased responsiveness to treatment, inability to achieve full recovery, and greater degrees of disability.^16^ ^,^^17^ Only one in seven patients experiences recovery when recovery is defined as a very good outcome in two domains—clinical and social/functional for at least two years.^18^

## Primary symptoms of schizophrenia

### Positive symptoms

Positive symptoms—hallucinations and delusions—refer to ideas, beliefs, and perceptions that *add to or distort* an individual’s normal functioning.

A delusion is a false belief, judgment, or knowledge that is held despite evidence to the contrary. The decision to call a belief a delusion is made by an observer, not by the believer, since the believer holds the delusional belief with the same conviction as nondelusional beliefs. A delusion transforms an individual’s basic experience of the world. For example, Marta’s experience of the world is one of malevolence and persecution. Twelve delusional themes have been identified with persecutory delusions being the most common with a pooled point prevalence of 64.5% (60.6-68.3), followed by referential delusions 39.7% (34.5-45.3), grandiose delusions 28.2% (24.8-31.0), delusions of control 21.6% (17.8-26.0), and religious delusions 18.3% (15.4-21.6).^19^ It is not uncommon for patients to have multiple delusions.^20^

Hallucinations are false perceptions that occur spontaneously in any sensory modality (sight, hearing, smell, taste, touch). Auditory hallucinations are the most common type of hallucinations with a lifetime prevalence of up to 80%.^21^ Auditory hallucinations can take the form of sounds (bells, screeches, screams) or voices (auditory verbal hallucinations, AVH). AVH can range from single words to conversations involving multiple different voices to voices giving commands. Visual hallucinations have been observed in up to 26% of patients, followed by tactile hallucinations, olfactory hallucinations, and gustatory hallucinations, which occur less frequently.^22^ ^-^^24^ Visual hallucinations have been associated with more severe illness, suicide attempts, and with certain types of delusions.^22^ ^,^^25^

Hallucinations and delusions may emerge concomitantly, one may emerge before the other, or only one or the other may be present.^26^ The formation and content are influenced by the social and cultural background of the patient.^27^ ^,^^28^ Positive symptoms typically follow a remitting and relapsing course.^29^ ^-^^31^ Persistent delusions and hallucinations can interfere with employment,^32^ relationships, daily function, and increase the risk of violence.^33^ Activity (as opposed to inactivity) and frequent social contacts are protective. Positive symptoms are the symptom cluster that responds best to currently available antipsychotic medications; however, the rate of response drops from 90% to 65% across the first two relapses.^34^

### Negative symptoms

Symptoms that are consistent with a deficit or loss of function are called *negative symptoms* and are apparent in two domains: expression (blunted affect, alogia) and motivation (amotivation, asociality, anhedonia). Negative symptoms commonly emerge during the prodromal phase and are a risk factor for progression to psychosis.^35^ In chronic schizophrenia, almost 60% of patients demonstrate at least one negative symptom with social withdrawal being the most common (45.8%), followed by emotional withdrawal (39.1%), poor rapport (35.8%), and blunted affect (33.1%).^36^ Negative symptoms worsen with age and are poorly responsive to most currently available pharmacologic treatments,^30^ ^,^^37^ although potential treatments may be on the horizon.^38^ Primary negative symptoms are directly related to the pathophysiology of the illness, while secondary negative symptoms are caused by antipsychotic side effects, comorbid depression, substance use, social deprivation, or as sequelae of positive symptoms.^39^ Since secondary negative symptoms can be treated, disentangling them from primary negative symptoms is important.

As shown in Table 3, negative symptoms significantly impact an individual’s ability to live independently, build relationships, and maintain employment. They are a key contributor to schizophrenia-related disability.^40^ ^-^^44^

**Table 3.** Negative Symptoms, Association with Other Impairments, and Functional Outcome

Domain	Characteristics^119^ ^,^^120^	Relationship to cognitive impairments and impact on functional outcomes
Expression		
Blunted affect	Components: 1. Decrease in facial expression and eye contact; 2. Decrease in expressive gestures and body language; 3. Decrease in vocal expression	Related to social cognitive domain of emotion processing, i.e., patients with flat affect show greater impairment in emotion processing^121^ Reduced facial expression predicts less favorable social interactions^122^ ^,^^123^ Reduced nonverbal behaviors are associated with poorer social outcomes^124^
Alogia (poverty of speech)	Decreased quantity of speech, reduced spontaneous speech, loss of conversational fluency, avoidance of communication	Related to cognitive domain of verbal fluency, i.e., as severity of alogia increases, verbal fluency decreases^125^ Lower levels of inattention-alogia predicted competitive employment two years after acute exacerbation^126^
Motivation		
Amotivation/avolition	Reduction in the desire to perform, initiate and maintain goal-directed activities such as work, studying, sports, and personal hygiene. Includes a subjective reduction in interests and desires and a behavioral reduction of self-initiated and purposeful acts	Mediates relationship between neurocognition and social cognition^41^ Social amotivation is negatively correlated with employment at one and two years after acute exacerbation^126^ Significant negative correlation with functional outcomes, e.g., interpersonal relations, social participation, recreation, self-reliance and execution, employment^44^
Asociality	Reduction in the frequency of social interactions, decreased interest in forming relationships with others. Loss of interest in intimate (sexual) relationships	Overlap with social cognition^68^ Significant negative correlation with functional outcomes, e.g., interpersonal relations, social participation, recreation, self-reliance and execution, employment^44^ Potentially linked to social disconnection which is associated with poorer physical health^68^
Anhedonia	Reduced ability to anticipate or experience pleasure across activity domains (e.g., social, physical, recreational, work/school). The anticipation of pleasure is more impaired than the ability to experience pleasure in the moment	Certain cognitive deficits are risk factors for anhedonia. Specifically, language deficits are a risk factor for anticipatory anhedonia, while delayed memory deficits are a risk factor for consummatory anhedonia^127^ Over the course of the illness, physical anhedonia becomes more strongly associated with worse functional status^128^

### Disorganization

Disorganization emerged as a symptom cluster when it separated from positive and negative symptoms in studies using factor analysis.^45^ ^-^^47^ Since an individual’s thought process cannot be known, it is inferred through communication or speech. Disorganized speech infers disorganization of the form of thoughts and reflects a cluster of related cognitive and linguistic disturbances.^48^ This became known over time as a *formal thought disorder* to distinguish it from pathology involving the content of thought, i.e., delusions and ideas of reference. Ozbek and Alptekin (2022) define a formal thought disorder as “…any deficiency of organizing words, concepts, phrases, or ideas in a logical order to express a certain purpose.”^49^ Disorganization is commonly identified in speech that seems to slip off track or derails, is tangential, or is circumstantial.^45^ More pathological forms of disorganization are incoherent speech, neologisms, clang association, thought blocking, and echolalia.

As an individual becomes increasingly psychotic, the degree of disorganization increases exponentially.^48^ Disorganization is a prominent feature in first-episode patients, and it diminishes with treatment.^50^ It affects approximately half of patients with schizophrenia.^51^ As expected, there is a strong relationship between disorganization and all domains of neurocognitive functioning^41^ and a significant, inverse relationship with social functioning.^52^

## Cognition in schizophrenia

Cognition encompasses neurocognition and social cognition. Neurocognition refers mental abilities such as attention, memory and learning, reasoning and problem-solving. Social cognition includes processes involved with perceiving, processing, and regulating information about other people and ourselves. Deficits in neurocognition and social cognition result in difficulties in social, educational, and occupational spheres of life.^1^ ^,^^2^ ^,^^4^ ^,^^5^ ^,^^32^ ^,^^53^ ^-^^55^

## Neurocognition in schizophrenia

There is considerable cognitive heterogeneity among individuals with schizophrenia. Three cognitive subgroups have been identified: a group that is relatively cognitive intact with mild impairments (25% of sample), an intermediate group, and a group with severe and widespread deficits (44% of the sample).^56^ The magnitude of the neurocognitive deficits can be significant with scores of 1-2 standard deviations below healthy controls across multiple domains.^57^ ^-^^59^

Evidence of neurocognitive impairments can be seen as early as childhood. Woodberry and colleagues (2008) found that years before the onset of psychotic symptoms, as a group, individuals with schizophrenia demonstrated mean IQ scores that were one-half standard deviation below those of healthy controls.^8^ Deficits in attention, memory, executive functions, and processing speed have been observed in the premorbid stage.^60^ Although data from shorter-term cohort studies suggests that cognition remains fairly stable after illness onset, Jonas and colleagues (2022) performed the largest long-term cohort study of cognition which showed that the trajectory of general cognitive ability is stable until 14 years prior to psychosis onset. Then, general cognitive ability declines from adolescence through the first psychotic episode and during the first two decades of the illness. At age 49, a second decline in cognitive abilities commences, well before that seen in healthy controls.^61^ ^,^^62^ This earlier decline may be due in part to accelerated aging.^63^

Neurocognitive impairment is correlated with functional disability (Table 4) and is responsible for the indirect costs of the disease. Typical goals of adulthood—educational achievement, competitive employment, self-sufficiency, self-care—may not be attained by individuals with schizophrenia due to cognitive impairments and social deficits. For example, at any given time, as few as 10% of individuals with schizophrenia are employed.^64^ Approximately 25-40% of people with schizophrenia live independently, and over 75% of this group is supported by disability compensation.^32^ Only 31% of people with schizophrenia own a car.^65^ Cognitive impairments interfere with patients’ ability to manage chronic medical conditions and medications. Medical problems go untreated, and poor medication adherence increases the risk of a psychotic relapse.^32^

**Table 4.** Profile of Neurocognitive Dysfunction in Schizophrenia and Real-World Example Using a Patient-Case

Domain	Description of domain	Degree of impairment	Real-world example of functional impairment
Attention	Selective attention is the ability to focus on relevant stimuli and ignore irrelevant stimuli. Sustained attention is the ability to maintain a consistent focus.	Moderate^129^	While in class, DeShawn is distracted from the lecture by the rustling of papers and the sounds outside. Even at home where it is quiet, he has trouble maintaining a consistent focus on what he’s reading.
Working memory	Verbal working memory is the ability to temporarily store verbal information in order to plan and carry out behavior. Visuospatial working memory is the ability to retain and process an object’s identity and location in space.	Moderate to severe^130^	In biology lab, the professor gave instructions for how to prepare a microscope slide. DeShawn struggled to remember the sequence even after the lab tech repeated it. DeShawn has practiced labeling the parts of a cell multiple times, so he remembers where they are for the test.
Processing speed	Processing speed is the time that it takes to perceive information, process it, and formulate or enact a response.	Moderate to severe^131^	In his college algebra class, DeShawn is the last student to hand in his test and he did not have enough time to work through all the problems.
Executive functions	Executive functions are a set of interrelated, higher level cognitive processes, and abilities that allow planning and execution of goals and monitoring behavior. Core subcomponents are response inhibition and interference control, working memory, and cognitive flexibility.	Moderate^132^	After school, DeShawn goes to the tutoring center for help with organizing the homework from his different classes, structuring his study time, and completing papers and projects on time.
Verbal fluency	Verbal fluency is the ability to move from thoughts to words and involves the capacity to access and organize vocabulary.	Mild-Moderate^133^	DeShawn’s small group collected differing viewpoints on a local water issue. Although DeShawn understood the project, when it came to interviewing members of the public, he struggled to formulate and ask the interview questions.

### Social cognitive impairment

Approximately 75% of people with schizophrenia demonstrate at least mild impairments in social cognition.^66^ Similar to neurocognitive deficits, there is considerable heterogeneity with most people experiencing mild-moderate deficits and one-third of patients suffering from severe impairments (Table 5). Severe impairments are associated with older age, fewer years of education, symptom burden, more neurocognitive impairment, and poorer function.^66^ ^,^^67^ Social cognitive impairments are present well before the onset of the first psychotic break and remain stable over the course of the illness.^1^ ^,^^53^ ^,^^68^

**Table 5.** Social Cognitive Processes and Real-World Example of Impairment Using Patient Case

Social cognitive process	Definition	Degree of impairment	Real-world example
Perception of social cues	Perceiving social cues contained in facial expressions, voices, and body movements allows appropriate responses that drive interpersonal interactions.	Significant^134^	At the motel where Marta lives, she observes the manager frowning as he enters the laundry room. Marta notices his pursed lips and wrinkled brow. Although he greets her with, “Good Morning, Marta!” his voice sounds irritable, and he is standing with his arms crossed over his chest. Marta interprets these social cues as indicating that the manager is angry with her.
Mental state attribution	Mental state attribution (MSA) is the ability to infer the mental state of others. Cognitive MSA is the capacity to interpret another person’s beliefs. Affective MSA is the capacity to interpret another person’s feelings	Significant impairment in both cognitive and affective MSA^134^	While Marta initially felt good to be greeted kindly, the feeling fades as she thinks about the manager’s frown. Marta infers that the manager believes that she is vandalizing the laundry room. She interprets his facial expression and body language as irritation and frustration with her.
Attribution bias	Attribution bias or style reflects how people explain their actions, the intentions and actions of others, and events.	Consistent impairment has not been demonstrated^134^ ^,^^135^	Marta wonders if the manager’s irritation with her is due to an episode months ago when a washer broke and flooded the laundry room.
Emotion processing	Emotion processing refers to an interrelated set of abilities that includes experiencing, understanding emotions, differentiating different emotions, and managing emotions.	Emotional experience is intact^53^ Significant impairment in other aspects of emotion processing^134^	Marta initially felt good about the manager’s greeting. Several hours later and as she ruminates about his frown, she becomes increasingly fearful that he is going to ask her to leave the motel.

Social cognition demonstrates an even stronger link to community functioning than does neurocognitive impairment and accounts for about 16% of the variance in functioning compared to 6% for neurocognitive functioning.^55^ Deficits in mental state attribution, or the ability to infer the mental state of another, account for most of the variance. Social disability encompasses three areas: independent living, education and employment, and interpersonal relationships. In terms of interpersonal relationships, friendship networks tend to be small (mean number of friends is 1.57) but when present, friendships are highly valued.^69^ Although approximately one-third of patients never marry, those who do find marriage to be a source of support.^70^ People with schizophrenia may experience social disconnection, which appears to be due to impairments in both social cognition as well as social motivation.^68^ Social disconnection is associated with an increased risk of adverse health outcomes and all-cause mortality.

## Accessory symptoms of schizophrenia

### Mood disturbances

Depression is common in all phases of schizophrenia with factor analysis identifying it as a major symptom dimension. Over 40% of help-seeking individuals at high risk for developing psychosis fulfill criteria for a depressive disorder.^71^ During the prodromal phase of the illness, over 60% of patients fulfilled criteria for a lifetime depressive disorder.^72^ Nearly 50% of first-episode patients have clinically relevant levels of depressive symptoms, with 25% experiencing a full-depressive episode.^73^ In chronic schizophrenia, higher rates of depression—up to 60% of patients—are seen during acute episodes^74^ compared to a rate of 30% during periods of stability.^75^ ^,^^76^

Depression is linked to negative outcomes in schizophrenia. It is a major risk factor for suicide and is present in over 50% of patients who die by suicide.^77^ Following a cohort of depressed patients with schizophrenia over three years, Connelly and colleagues (2007) found that depressed patients were more likely to suffer a relapse of psychosis, use substances, be a safety concern (violent, arrested, victimized, suicidal), report poorer relationships and greater functional impairment, suffer from poorer health (mental, physical), and be less adherent with treatment.^78^ Despite the high prevalence and association with negative outcomes, major depressive disorder in schizophrenia is underdiagnosed. Moreover, when diagnosed and treated with antidepressants, 44% of patients remain symptomatic.^76^

The diagnosis of depression in schizophrenia is not straightforward. First, there is a significant overlap between depressive symptoms and other symptom clusters. For example, anhedonia, apathy, and social withdrawal are negative symptoms and are seen in depression. Cognitive dysfunction is common to both depression and schizophrenia. Antipsychotic treatments and their effects on dopaminergic neurotransmission can produce drug-induced parkinsonism, which may be associated with anergia and emotional withdrawal or akathisia, which is associated with dysphoria. Patients who use illicit substances may experience dysphoria during substance withdrawal.

### Anxiety

In parallel with what is observed for depression, anxiety symptoms and syndromes are common in all phases of schizophrenia with rates several-fold that seen in the general population. They contribute to negative outcomes^79^ ^,^^80^ and are underrecognized.^81^ In terms of prevalence, 38.5% of schizophrenia patients have at least one comorbid anxiety disorder with 14.9% fulfilling criteria for social anxiety disorder, 12.4% for post-traumatic stress disorder (PTSD), 12.2% for obsessive-compulsive disorder (OCD), and 10.9% for generalized anxiety disorder, followed by panic disorder and specific phobia.^82^ Although the DSM-5-TR has reconceptualized PTSD as a trauma and stressor-related disorder as opposed to an anxiety disorder, it shares neurobiological features with anxiety disorders.^83^

### Violence

Although most patients with schizophrenia do not engage in violent behavior, schizophrenia increases the risk for violence.^84^ ^-^^87^ Short et al (2013) used a case linkage design to compare patterns of violence between 4168 schizophrenia patients and community controls. Of the schizophrenia sample, one in four patients had been charged with a criminal offense, and one in ten had been convicted, a rate higher than that seen in the community sample (10% of people charged, 2.4% convicted).^85^ Substance use disorders (SUDs) increase risk; however, violence cannot be entirely attributed to substance use.^85^ ^,^^88^ Women with schizophrenia are at greater risk of committing a violent act than men with schizophrenia.^84^ ^,^^85^ Victims of violence are acquaintances (49.7%), followed by relatives (28.9%), and strangers (21.4%).^89^ Among relatives, mothers of individuals with schizophrenia are the most common target of violent acts and threats.^90^ Many factors have the potential to increase the risk of violence in schizophrenia. These include the catechol-*O*-methyltransferase genotype, developmental factors (childhood trauma, conduct disorders), antisocial personality disorder, substance use, neurocognitive impairment, treatment nonadherence, and certain positive symptoms such as persecutory delusions.^91^ ^,^^92^

## Comorbidities in schizophrenia

### Substance use

Substance use often predates the first psychotic episode with half of first-episode patients fulfilling criteria for a co-occurring SUD.^93^ Cannabis exerts a dose-dependent effect on the risk of developing a psychotic illness and accelerates illness onset by almost three years in regular users compared to non-users.^94^ ^,^^95^ Methamphetamine appears to increase the risk of developing schizophrenia on par with cannabis.^96^ In patients with an established illness, 50% have a lifetime history of a SUD, a rate five-times greater than that in the general population.^97^ ^,^^98^ Alcohol is the most used substance, followed by cannabis, and then followed by other drugs.^99^ ^,^^100^ At any timepoint in the illness, SUDs are associated with greater positive symptom severity, less treatment adherence, more aggression and violence, and poorer social functioning.^101^ Several different hypotheses attempt to explain the high prevalence of SUDs in people with schizophrenia. These include the *primary addiction hypothesis*, which proposes that substance use and schizophrenia share abnormalities in striatal dopaminergic neurotransmission and the *two-hit model* in which substance use is an environmental stressor that precipitates the development of psychosis in vulnerable individuals.^100^ ^-^^103^

### Suicide

Psychotic disorders have one of the highest rates of mortality among mental disorders.^104^ Suicide is the greatest relative risk factor for mortality in people with schizophrenia.^105^ Using linked national databases to follow approximately 76,000 people with schizophrenia for up to 20 years, Zahar and colleagues (2020) found that 1 in 58 individuals died by suicide, with suicide typically occurring within 4 years of the initial diagnosis.^106^ Further, 25-50% of patients attempt suicide^107^ for an overall increase of 50-100-fold compared to that of the general population.^108^

Popovic and colleagues (2015) performed a systematic review of 77 studies to identify risk factors for suicide with the most conclusive evidence base. Factors that were strongly associated with suicide in hospitalized patients and outpatients were depressed mood, history of suicide attempt(s), and the number of psychiatric hospitalizations. Other factors included hopelessness, younger age, close proximity to illness onset, and hospital admission (during admission or within one week of discharge). While male gender and substance use have been associated with suicide in the general population, the data in schizophrenia are mixed.^77^ Command auditory hallucinations often command self-harm or suicide; however, the data have not shown them to be a consistent risk factor for suicide.^109^ Nevertheless, this type of auditory verbal hallucination should prompt careful suicide risk assessment and safety planning. The primary protective factor against suicide is adherence to comprehensive treatment including pharmacotherapy and psychosocial treatments.^105^ ^,^^109^ ^,^^110^

## Conclusion

**Figure 2.**
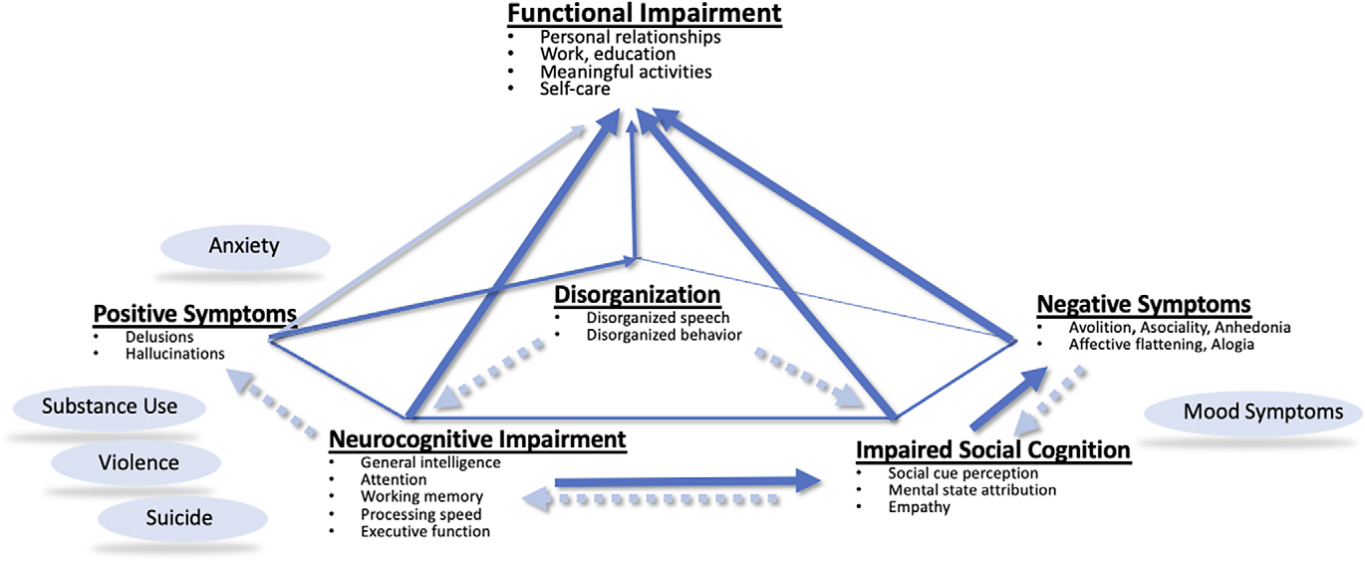
The relationship between core schizophrenia symptoms and functional impairment. Core symptoms affect each other to varying degrees. For example, there is a strong relationship between disorganization and all domains of neurocognitive functioning and an inverse relationship with social functioning. Negative symptoms are associated with deficits in empathy and the ability to infer emotions, impacting social cognition. Neurocognitive and social cognitive deficits along with negative symptoms have the greatest impact on functional outcome.

The symptoms and signs of schizophrenia and schizophrenia-spectrum disorders are well established. The patient cases—Marta and DeShawn—illustrate the heterogeneity in the clinical presentation of schizophrenia, the disease trajectory, and in functional outcomes. While Marta’s hallucinations, delusions, and disorganization or DeShawn’s asociality and alogia are obvious, both patients suffer from neurocognitive and social cognitive impairments that impact their ability to engage in meaningful activities, manage independent living, and navigate productive interactions with others. Figure 2 illustrates the relationship between schizophrenia symptom clusters and functional impairments.

These patients’ experience confirms that treatment of positive symptoms is the first step in improving function.^5^ ^,^^32^ ^,^^33^ ^,^^110^ ^,^^111^ There’s a clear relationship between Marta’s persecutory delusions and violence. When her illness is optimally treated with antipsychotic medication, the conviction with which she holds delusional beliefs diminishes, her perception of others as malevolent dissipates, and violence risk decreases, outcomes described in the literature.^112^ ^-^^114^ For Deshawn, pharmacologic treatment of positive symptoms after the first psychotic break resolved suicidality and prevented rehospitalization. Treatment at the time of the first psychotic episode is critical to improving symptomatic and functional recovery.^14^ ^,^^34^ ^,^^105^ ^,^^115^ ^-^^118^ Psychiatric stability allowed Deshawn to resume his education. The next step in treatment is implementing psychosocial treatment for negative symptoms, neurocognitive symptoms, and social cognitive symptoms, with the goal of improving interpersonal relationships and facilitating academic and occupational success.
